# Weeds as Pathogen Hosts and Disease Risk for Crops in the Wake of a Reduced Use of Herbicides: Evidence from Yam (*Dioscorea alata*) Fields and *Colletotrichum* Pathogens in the Tropics

**DOI:** 10.3390/jof7040283

**Published:** 2021-04-09

**Authors:** Pauline Dentika, Harry Ozier-Lafontaine, Laurent Penet

**Affiliations:** UR ASTRO, INRAE, F-97170 Petit-Bourg, Guadeloupe, France; Pauline.dentika@inrae.fr (P.D.); harry.ozier-lafontaine@inrae.fr (H.O.-L.)

**Keywords:** *Colletotrichum gloeosporioides*, anthracnose, *Colletotrichum truncatum*, crop disease, water yam, *Dioscorea alata*, weeds, selective weeding

## Abstract

The transition toward sustainable agriculture requires rethinking cropping systems in the light of less intensive and chemically reliant practices. Weed management is one of the target practices to evolve cropping systems with decreased impact on the environment. While softened management will lead to increased weeds/crops coexistence, it is of importance to assess the relative benefits and drawbacks of new practices. Among the potential drawbacks of weeds/crops coexistence, disease risk may increase if weeds are hosting pathogens. In this study, we assessed the potential of weeds for hosting pathogenic generalist fungi known to translate into disease in crops. We first describe prevalence in fields after harvest and relate prevalence to species characteristics and communities. Then, we directly test the idea that weeds serve as inoculums sources during cropping with a natural experiment. This study highlights variation in host skill among feral weeds for *Colletotrichum* species, including potential congeneric sub-specialization on different weeds within communities. Last, prevalence within fields was more correlated to focal crop inoculation rates compared to local weed load, but there was a significant correlation effect with prevalence on weeds in the vicinity of fields, suggesting that weeds are mediating disease levels at the local scale, too. Results pointed to the importance of weed host skill in disease risk yet open the door to the potential control of pathogens via targeted weed management.

## 1. Introduction

There is a global increased awareness of the negative impacts of intensive agriculture, among which concerns about environmental degradation due to the overuse of synthetic chemicals [1], carbon dioxide or greenhouse gas effects [2], and biodiversity rarefaction [3]. These issues are opening a wide range of questions as to how new sets of practices and cropping systems might both maintain productivity levels and mitigate detrimental consequences of intensification [4]. Among targets of potential improvement toward increased sustainability, a focus on weed management strategies would allow a diverse array of actions [5], depending on the consequences of more flexible options regarding weed/crop coexistence in the fields.

Weeds are traditionally mostly seen as direct crop competitors for resources [6], including access to light, water, and soil nutrients. These factors might actually be rather species-specific and relative to the natural plant communities [7] and potential misfit of crops within these assemblages (although sometimes, crop competitiveness is also used to control for weeds, e.g., [8]). Thus, relaxing weed control under this view would translate in increased competition and thus lower yields in crops. Little is known regarding which component of weeds/crop competition is most likely having an impact on yield (but see [9]), especially since the relative importance of these components might vary during seasons or development stages of crop plants or both (e.g., competition for light is critical when crops are at emergence and seedling stages, while competition for nutrients might have greater impact during growth and competition for water during the main photosynthetic stage, including producing tubers or seeds, e.g., [10,11,12]). Since diversity in practice is affecting weed communities [13], the road toward sustainable agriculture should be paved with a specific set of practices on par with constraints on productivity [14].

On the other hand, several ecological processes might compensate for the negative impact of increased competition. For example, increased diversity at field edges is known to provide shelters for entomological fauna with positive impact against crop antagonists (e.g., effect of increased demography of parasitoids) or increased pollination services [15]. Other indirect effects are also often overlooked, such as soil-mediated interaction effects between plant species, often providing yield support and considered as the main driver of intercropping advantage [16]. Positive ecological interactions are indeed often an overlooked component of yield success [9], yet they sometimes account for an important share of productivity. Weeds might also contribute to the numerous ways in which positive ecological interactions account for field productivity [15,17], from attracting beneficials, deterring antagonists, and even underground effects such as soil community feedbacks including help to mycorhization [18]. In addition to competition effects and beneficial interactions, other negative impacts are often overlooked too, such as the potential of weed species to host pathogens [19], leading to potentially increased disease risk if coexistence with weeds is increased. Most pathogens are often specific in the range of plants they attack, so the issue becomes mostly a question of phylogenetic inertia of shared pathogen load [20], *i.e.*, it revolves mostly around weeds and crops that belong to closely related botanical families. Nevertheless, the effect might not be negligible for broad range pathogens, i.e., those demonstrating a high degree of generalism.

In this study, we investigated prevalence in weeds of the fungus responsible for anthracnose on Water Yams (*Dioscorea alata*), namely *Colletotrichum gloeosporioides*, and its congeneric alternative, *C. truncatum* (a fungus casually found in yams, without drastic effect on crop health). *Colletotrichum* fungi are indeed quite generalist and known to occur in weed species (e.g., [21], including *C. acutatum* [22]). *Colletotrichum gloeosporioides* causes anthracnose in Yams and poses serious threat to yield [23,24], spreading locally via rain splash [25] and possibly initiating disease via tuber seed contamination [26]. Pathogenic strains are known to be possibly hosted on a broad array of potential hosts [27]. Yam crop demonstrates strong sensitivity to variation in cropping system (e.g., [28,29]). Anthracnose disease even had a demonstrated effect on management by producers and varietal diversity [30,31]. In a first step, we describe weed species at higher risk of causing a threat to the crop because of their host relationship with *C. gloeosporioides* and test whether morphological characteristics were associated with either fungus. In a second step, we analyzed whether weed communities were indeed increasing pathogen prevalence in yam fields. We discuss our results in the light of potential control strategies for weeds, especially practices focusing on targeted weed species management.

## 2. Materials and Methods

### 2.1. Prevalence of Colletotrichum on Feral Weeds

In a first step, we investigated weed species diversity in three previously cultivated yam plots (two had been just harvested, and one was cultivated the previous year) in 2018. We collected weed samples from three post-harvest yam fields at INRAE institute at Duclos (Guadeloupe) (central coordinates ‘16.2023, −61.663105’ for neighboring fields and ‘16.201705, −61.661414’, altitude ca. 101 to 107.1 m above sea level). Local flora at the site was typical of the region (all identified species are common feral weeds in the area and all were also found in the farmers fields). Every weed species, save those belonging to the Monocot clade, were recorded and identified to species level (save 4 species that were only identified to genus level). Then, we harvested randomly one leaf for each species up to ten different individuals within species, thus in total a grand total of 174 samples for strain isolation. Sampled leaves were picked in the fields and immediately placed in plastic bags and labeled before leaving the bags in a refrigerated cooler box until field sampling was completed.

Leaves were brought back in the lab for strain isolation, where they were washed in 4 successive baths of 30 s each, first in a 10% diluted bleach solution, then rinsed in water, then a methanol bath, and eventually a last rinsing step in water. Further work was done in sterile conditions under a Laminar flow cabinet (model LRF 48). Leaf pieces were cut and placed on Petri dishes with S medium [Ca(NO_3_)_2_ 10 g·L^−1^ + KNO_3_ 2.5 g·L^−1^ + MgSO_4_ 2.5 g·L^−1^ + KH_2_PO_4_/K_2_HPO_4_ 5 g·L^−1^ + saccharose 5 g·L^−1^ + malt 1 g·L^−1^ + citric acid 50 mg·L^−1^ + Agar 25 g·L^−1^] to select for and positively enhance *Colletotrichum* species, and sealed with parafilm tape following our routine lab procedures. After an incubation period of 4 to 6 days under 12 h light (under Osram T8 L 36 W/865 Lumilux Daylight G13 neons, similar to daylight) at room temperature (22–28 °C), conidia from the Petri dishes were observed under a light microscope for species identification based on spore morphology [32], and to estimate *Colletotrichum gloeosporioides* and *C. truncatum* prevalence in the different species in the fields.

### 2.2. Plant Characteristics Associated with Prevalence

In parallel to strain isolation, we compiled a matrix of weed species morphological and habit characteristics based on Fournet Flora [33], either with quantitative estimates (or ranges, accounted for as two covariates—minimal and maximal character values) or binary character states. Prior to analysis, we discarded factors from the matrix for which variation threshold was less than at least one-fourth of the species presenting the less common variation. Thus, we recorded the 24 following characteristics for every weed species recorded in the fields (save the four unidentified species): erect (yes or no), minimum height (in cm), maximum height (in cm), composite leaf (yes or no), ovate (yes or no), oblong (yes or no), lanceolate (yes or no), hairy leaf (yes or no), opposite leaves (yes or no), obtuse (yes or no), cuneiform (yes or no), united veins (yes or no), alternate leaves (yes or no), pinnate leaves (yes or no), entire leaf (yes or no), serrate leaves (yes or no), climbing habit (yes or no), creeping habit (yes or no), minimum petiole length (in cm), maximum petiole length (in cm), petiolate (yes or no), sessile leaf (yes or no), prevalence of Colletotrichum gloeosporioides (ratio, no unit), prevalence of Colletotrichum truncatum (ratio, no unit). Binary characteristics were expressed as 0 (lacking) or 1 (possessing) the feature.

### 2.3. Experimental Field Study of Coinfection between Yams and Weeds

In a second step, we focused on weed species that demonstrated the highest prevalence in yam fields and yam plants of the same area. Experimental sampling occurred in typical vegetation time in the middle of rain season (October to November) in 2019. Unfortunate events prevented replication of the study the previous and subsequent years (social unrest and road traffic issues and cropping disruptions from Covid pandemics). We followed the same isolation protocol as described previously. For each sampled field, we decided to divide the cultivated area in quarters in order to account for heterogeneity in local weed communities. Eight distant yam plants were sampled for *Colletotrichum* in each quarter. Up to 6 leaves on individual weeds for every “high prevalence” species (prevalence >50%, see Table 1) were sampled and checked for *Colletotrichum* for every quarter of a field, and in the vicinity within 5 m ahead of the field edge. Thus, prevalence in *Colletotrichum* species were estimated for both yam plants from focal quarter, yam plants in the remaining of the field, weeds within the focal quarter, and weeds in the vicinity of the field.

In total, ten farmers accepted that we sampled for disease in their fields reflecting globally a diversity of situations typical of the region (Basse Terre, Guadeloupe, the production basin for yams), although two fields were harvested before sampling was completed, and two producers eventually retracted. The fields that were the focus of the study were respectively located at Blonzac (2 fields, coordinates ‘16.1436, −61.62321’ and ‘16.143471, −61.623041’), Convenance (2 fields, coordinates ‘16.243254, −61.592158’ and ‘16.243368, −61.592362’), Barthélémy (1 field, coordinates ‘16.116547, −61.589592’), and the agronomic plot at INRAE institute mentioned above (coordinates ‘16.201705, −61.661414’). All fields sampled in this study were fallows prior to yam cropping and have been tilled and traditionally organized in ridges before plantation. The crops were grown on typical Guadeloupean ferralsols, with potentially little variation of acidity [34]. *Colletotrichum* fungi are not able to survive in soils [35] and are assumed to contaminate fields either from local vegetation inoculums or possible via infected tuber seeds [26]. *Dioscorea alata* is a genetically highly diverse species [36], and varieties growing in Guadeloupe reflect this diversity as new varieties from worldwide origin were proposed for their natural resistance to anthracnose disease in the 70s. Thus, fields were planted with diverse varieties in admixtures, or quasi monocultures of frequent varieties, and all included the following varieties (Kabusah, Pacala, Goana), from which our samples were collected. A grand total of 192 yam leaves and 864 weed leaves were sampled to assess prevalence of *Colletotrichum* species during yam cultivation.

### 2.4. Statistical Analyses

We analyzed data with R software [37], first describing weed diversity based on the morphology and prevalence via a Principal Component Analysis, in order to investigate potential relationships between infection skills of both *Colletotrichum* species and descriptive morphological and habit covariates. In the second approach, we run an ANOVA with focal prevalence on Yams as the dependent, and prevalence on yams in the remaining of the field, prevalence on weed within field, and prevalence on weed in the immediate vicinity of the field as independents, and interactions between these factors. We used prevalence estimates rather than absolute number of strains that were isolated, to account for differences in sampling effort between focal area and estimates within fields and vicinity (calculated as the sum of surrounding local prevalences). In the ANOVA, conditions of homogeneity of variances and normality of residuals were met.

## 3. Results

### 3.1. Prevalence of Colletotrichum on Feral Weeds

A total of 31 weeds species were identified in four previously cultivated yam fields, belonging to 15 botanical families (Table 1). Mean prevalence on weeds was 35% and 24% for *C. gloeosporioides* and *C. truncatum* respectively, with a range of 0–80% within post-harvest fields for both. Ten species were identified as demonstrating a high prevalence of *Colleotrichum gloeosporioides* and thus presenting potentially a higher risk of increasing anthracnose disease on yams (Table 1): *Alocasia macrorrhiza*, *Bidens alba*, *Datura stramonium*, *Indigofera* sp. and *Indigofera spicata*, *Malachra fasciata*, *Passiflora* sp. and *Passiflora foetida*, *Sida rhombifolia*, *Spigelia anthelmia*.

### 3.2. Plant Characteristics Associated with Prevalence

**Figure 1:**
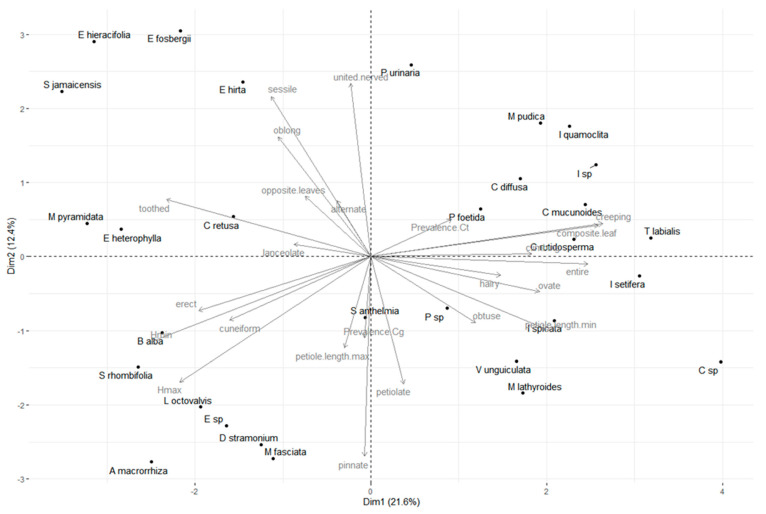
Principal Component Analysis of weed morphospace and prevalence of *Colletotrichum gloeosporioides* and *C. truncatum* in post-harvest Yam fields. First axis of PCA retains 21.6% of variance and second axis 12.4%. Morphological characteristics vectors are indicated in grey.

The two first components of PCA accounted for 34% of variance in the morphological diversity of weeds. Weed species were broadly interspersed with low aggregation levels, which is a result typical of high dimension morphospaces (Figure 1). The prevalence of both *Colletotrichum* species were negatively correlated and nearly antinomic (or at least negatively correlated), suggesting coexistence in the fields does not translate as a coexistence within plants but rather leads to a pattern of subspecialization on different weed species in the weed communities. *C. Truncatum* tends to be found on species with crawling characteristics at greater rates (vines or climbing plants, such as *Ipomea* and *Passiflora* for example), while *C. Gloeosporioides* was more often associated with species presenting longer petioles and pinnate leaves and generally stemming higher in the herbaceous canopy. Therefore, we can assume the spatial segregation of these *Colletotrichum* species onto different weeds at the local level.

### 3.3. Experimental Field Study of Coinfection Between Yams and Weeds

In our ANOVA analysis, focal prevalence of *Colletotrichum gloeosporioides* on yams was significantly impacted by prevalence on yams in the remaining of the field (Table 2), but prevalence of the fungus on weeds within fields was only marginally relevant to focal yams prevalence, and prevalence from weeds in the vicinity did not significantly impact inoculation levels on focal yams. On the other hand, there was a significant interaction between prevalence on yams in the remaining of the field and prevalence on weeds in the vicinity of the field on the onset of inoculation of focal yams (Table 2). Our results thus suggest that while inoculation is mainly dependent on disease onset in the remaining of the field, weeds are having a small effect as host relays at local level (within fields) and are mediating inoculation intensity at greater scales (vicinity of fields).

## 4. Discussion

We first described that *Colletotrichum gloeosporioides* (and *C. truncatum* respectively) can infect many weed species in the fields, and even coexist sympatrically at local scale (a phenomena already described in literature (e.g., [21,22]). They nevertheless showed potential for subspecialization toward preferential hosts, with *C. truncatum* demonstrating a tendency to occur on plants with creeping, crawling and climbing habits (liana, vines, etc.) and *C. gloeosporioides* on plants erect. Prevalence was high enough: mean prevalence on weeds of 35% and 24% for *C. gloeosporioides* and *C. truncatum* respectively (with range 0–80% for both); and mean prevalence 25.7% and 4.2% on yams respectively (with range 0–68.7% and 0–37.5% respectively). These prevalence rates are suggesting high potential for weeds as inoculums sources and disease start. These results were confirmed by the second experiment (Table 2), though prevalence on crop itself is the main driver of local epidemics, possibly as a filter effect for yam adapted strains. Weeds may nevertheless play an important role in disease initiation [38], especially since they are still present in the fields while yam crop is not after harvest. They seem to relay disease via host skill both at local level (within fields) and increase prevalence via interaction with crops at greater scales (vicinity of fields). We will discuss these findings in the light of current questions around decrease in herbicide use, and the potential of selective weeding.

Prevalence dynamics was more directly impacted by disease levels on crops within fields than by local weeds (Table 2), though weeds may mediate pathogen persistence during intercrop and thus play an important role as inoculums source. This would be especially expected if pathogen diversity is such that filtering effects existed, in that ‘relay role’ would be indiscriminate or bias strain infectious skills independently of their ability to harm specific crops. Indeed, diversity in weeds would translate in diversity in strains available, provided asymmetry in asexual reproduction on different host plants. On the other hand, both marginally significant effect of local weeds and interaction between local yam infection and weeds in the vicinity are pointing to an indirect role of weeds in disease starts. This means even a broad array of strains with diverse infection skills on crops might still translate in increased pathogen prevalence once the right strains reach crops and begin to multiply locally. Ideally, sustainable disease control strategies would be more efficient if they reduced the potential for filtering effects by weeds and thus have to decrease propagule load in the fields and in the local neighboring environment (see [39]).

There was variation in host competence for botanical families. Interestingly, the impacting antagonist, *C. gloeosporioides*, is found more often on plants that do not share the set of characteristics that *Dioscorea alata* possesses (e.g., it is a vine). Diversity in host skill in weeds also means that specific weed community composition might alter disease risk, and control strategies focusing on plants with higher host skill might decrease inoculums pressure during both intercropping and cultivation period (“alternate host suppression strategy”, see [40]). Less favorable weed communities might indeed decrease local propagule production (conidia) and inoculation rates in crops, and selective weeding might harness this potential as a disease control strategy. Nevertheless, this avenue of research is little explored currently, but it may prove an interesting path to more sustainable practices [5].

Both *Colletotrichum* species in this study demonstrated a tendency to subspecialize at the local scale on species with contrasting characteristics, and they were seldom sampled simultaneously within a host. This is suggesting competition for establishing successfully within hosts. Since mostly *C. gloeosporioides* is known to produce anthracnose disease in yams, it would be interesting to test whether these congenerics indeed compete locally and whether this could somehow leverage risks of epidemics in the crop. Diversity in host skill by weeds and potential for congenerics competition are opening the door to the possibility of selective (targeted) weed management via seed reduction [40]. While a weed management scheme based on targeted species erasure might both require botanical knowledge and plausibly increase labor load, disease risk might significantly decrease if high prevalence host species are preferentially eliminated from the fields. Increasing frequency of weeds more likely to host *C. truncatum* may also increase competition at the colonization stage for *C. gloeosporioides* and reduce its prevalence and thus risk for local epidemics. While there was a pattern suggesting antagonist presence between the congenerics (data not shown), data from this study were not amenable to test this hypothesis properly, and further evidence is required before a firmer conclusion could be reached.

## 5. Conclusions

In conclusion, it is theoretically possible to take advantage of diversity in host skill in weeds. By adopting a targeted weeding strategy focusing on feral plants with the highest prevalence levels for *Colletotrichum gloeosporioides*, it might be possible to decrease disease risk on yams. Selective weeding might also theoretically provide opportunities for increasing competition in the fields with its congeneric *C. truncatum*. Indeed, *C. truncatum* is not considered an efficient pathogen on yam crops, and the competition would further decrease opportunities for *C. gloeosporioides* to develop into an epiphytotic disease locally. While a targeted weeding management scheme would possibly increase the workload, its benefit would be a greater sustainability via a lessened reliance on chemicals. Local Caribbean species for which caution regarding increased anthracnose risk is warranted are *Alocasia macrorrhiza*, *Bidens alba*, *Datura stramonium*, *Indigofera* sp. and *Indigofera spicata*, *Malachra fasciata*, *Passiflora* sp., and *Passiflora foetida*, *Sida rhombifolia*, *Spigelia anthelmia*.

## Figures and Tables

**Table 1:** Listings of common weed species from Guadeloupean Yam fields by botanical families and estimated prevalence of *Colletotrichum gloeosporioides* and *C. truncatum* on these weeds in four post-harvest fields.

Weed Species	Botanical Family	Prevalence of *C. gloeosporioides*	Prevalence of *C. truncatum*
*Alocasia macrorrhiza*	Araceae	**0.8** *	0
*Bidens alba*	Asteraceae	**0.6** *	0
*Emilia fosbergii*	Asteraceae	0.17	0.08
*Erechtites hieracifolia*	Asteraceae	0.4	0.2
*Cleome rutidosperma*	Capparidaceae	0.2	0
*Commelina diffusa*	Commelinaceae	0.2	0
*Ipomea ipocea*	Convolvulaceae	0	0
*Ipomea quamoclit*	Convolvulaceae	0.2	0.2
*Ipomea setifera*	Convolvulaceae	0.4	**0.8**
*Euphorbia heterophylla*	Euphorbiaceae	0.2	0
*Chamaesyce Hirta*	Euphorbiaceae	0.4	0.2
*Euphorbia* sp.	Euphorbiaceae	0	0
*Phyllanthus urinaria*	Euphorbiaceae	0.2	**0.8**
*Calopogonium mucunoides*	Fabaceae	0	0
*Canavalia esculenta*	Fabaceae	0	0
*Crotalaria retusa*	Fabaceae	0	0
*Indigofera* sp.	Fabaceae	0.8 *	**0.8**
*Indigofera spicata*	Fabaceae	**0.6** *	**0.6**
*Macroptilium lathyroides*	Fabaceae	0	0
*Teramnus labialis*	Fabaceae	0.4	0.4
*Vigna unguiculata*	Fabaceae	0.4	0
*Spigelia anthelmia*	Loganiaceae	**0.8** *	0.4
*Malachra fasciata*	Malvaceae	**0.6** *	0.2
*Sida rhombifolia*	Malvaceae	**0.6** *	**0.6**
*Mimosa pudica*	Mimosaceae	0.2	0
*Ludwigia octovalvis*	Oenotheraceae	0.4	0.2
*Passiflora* sp.	Passifloraceae	**0.8** *	**0.8**
*Passiflora foetida*	Passifloraceae	**0.6** *	**0.6**
*Datura stramonium*	Solanaceae	**0.6** *	0
*Melochia pyramidata*	Sterculiaceae	0	0
*Stachytarfeta jamaicensis*	Verbenaceae	0	0.2

In bold, high prevalence levels recorded. Asterisks (*) are marking weed species presenting higher risk of increasing anthracnose disease due to prevalence of *Colleotrichum gloeosporioides*. These were also species specially targeted for sampling in the field experiment.

**Table 2:** Impact of local environment diversity in prevalence on inoculation rates of *Colletotrichum gloeosporioides* on focal yams. In bold, covariates with * *p* < 0.05; in italics, marginally significant covariates.

Dependent (Pathogen Source)	Sum of Squares	Df	F Value	*p*-Value
**local yams**	**3413.1**	**1**	**4.4882**	**0.0483** *
*local weeds*	*3014.2*	1	*3.9637*	*0.0618*
weeds in vinicity	380.2	1	0.5000	0.4885
local yams x local weeds	70.6	1	0.0928	0.7641
**local yams** **x weeds (vinicity)**	**3404.2**	**1**	**4.4766**	**0.0485** *

In bold, covariates with * *p* < 0.05; in italics, marginally significant covariates.

## Data Availability

The data presented in this study are available on request from the corresponding author.
